# Ruxolitinib plus extracorporeal photopheresis (ECP) for steroid refractory acute graft-versus-host disease of lower GI-tract after allogeneic stem cell transplantation leads to increased regulatory T cell level

**DOI:** 10.1038/s41409-020-0952-z

**Published:** 2020-05-23

**Authors:** Franziska Modemann, Francis Ayuk, Christine Wolschke, Maximilian Christopeit, Dietlinde Janson, Ute-Marie von Pein, Nicolaus Kröger

**Affiliations:** 1 Department of Stem Cell Transplantation, University Medical Center Hamburg-Eppendorf, Hamburg, Germany; 2 Department of Oncology and Hematology, University Medical Center Hamburg-Eppendorf, Hamburg, Germany

**Keywords:** Stem-cell research, Disease-free survival

## Abstract

Acute graft-versus-host disease (aGVHD) is a serious complication after stem cell transplantation and is associated with high non-relapse mortality. If steroid treatment as first-line therapeutic approach fails, treatment options are limited. In retrospective studies, ruxolitinib, a selective Janus kinase 1/2 inhibitor as well as extracorporeal photopheresis (ECP) could show high efficacy in treatment of steroid refractory acute and chronic GVHD. Here, we report single-center experience of combining JAK-inhibitor treatment with ECP in 18 patients with severe steroid refractory aGVHD of lower GI-tract. The treatment was well tolerated and no severe cytopenia (grade IV) occurred, in three patients grade III cytopenia could be observed. Response was complete or partial in 44% and 11%, respectively, resulting in an estimated 2 year overall survival of 56%. Steroids were tapered rapidly with a median time of 2 days for halving of dosage avoiding additional steroid-associated side effects. Under treatment with ruxolitinib and ECP, an increased level of regulatory T cells could be observed elucidating direct effects of this treatment on immune response.

## Introduction

Acute graft-versus-host disease (aGVHD) is a frequent complication and the major cause of non-relapse morbidity and mortality after allogeneic stem cell transplantation (ASCT). Even though the survival rates after ASCT have significantly increased over the past decades, the rate of developing aGVHD remains high with an occurrence in ~50% of all ASCT cases [1]. Main underlying pathology of this life-threatening complication is described by an inflammatory response and a disrupted host immune system caused by donor T lymphocytes, which are activated by host antigen-presenting cells [2]. Predominantly affected organs of aGVHD are upper and lower gastrointestinal tract (GI-tract), skin and liver. Nowadays, for prophylaxis and treatment of aGVHD, different strategies are available depending on severity (grades I–IV) of aGVHD or personal risk factors such as donor source or underlying disease. Standard therapy for aGVHD is 2 mg methylprednisolone per kilogram bodyweight, but only about 30–40% will respond to steroids with long durable remission [3, 4]. Outcome of steroid refractory aGVHD remains poor and no standard second-line therapy exists. Due to limited efficacy of second- and third-line therapies in these cases, new approaches are needed.

Ruxolitinib is a selective, small molecule Janus kinase (JAK) 1/2 inhibitor approved for therapy of advanced myelofibrosis and for polycythemia vera patients with inadequate response to hydroxyurea. Ruxolitinib causes a blockade of the JAK-STAT pathway which is, among a lot of other effects, known to play a role in T effector cell responses [5, 6]. Interestingly, it could be observed that among those patients who received ruxolitinib in myelofibrosis and respond to this treatment, a significant reduction of those plasma cytokines was measured, which play a major role in pathophysiology of aGVHD [7–9]. Furthermore, GVHD-mouse models and in vitro data underlined these findings and elucidated different potential mode of actions of JAK-STAT pathway in aGVHD [10]. In 2015, a clinical multicenter survey was reported by Zeiser et al.: 54 patients who received ruxolitinib in steroid refractory aGVHD and 41 patients who received ruxolitinib in steroid refractory chronic GVHD (cGVHD) showed an encouraging overall response rate (ORR) of 82% and 85%, respectively, with low relapse rates of 7% and 6% [11]. Based on these findings, an ongoing open-label, multicenter phase III clinical study was started (REACH2, NCT02913261) in 2016 in Europe, comparing ruxolitinib therapy versus best available treatment in steroid refractory aGVHD after ASCT. Recently, ruxolitinib has been approved by the Food and Drug Administration in the US for therapy of steroid refractory acute GVHD.

Main severe side effects of ruxolitinib therapy, particularly in combination with other immunosuppressive therapies, are prolonged pancytopenia attended by severe infections and bleeding complications [12].

Another therapeutical approach for aGVHD and cGVHD is extracorporeal photopheresis (ECP) using UV-A light in combination with 8-methoxypsoralen to induce apoptosis of leukapheresis gained mononuclear cells. Safety and efficacy of ECP administered in 21 patients with aGVHD and 88 patients with cGVHD as second- or third-line treatment in GVHD were reported recently. ORR in aGVHD was 84% [13].

Here, we investigated a single-center experience of the tolerability and efficacy of combining both therapeutic strategies—treatment with ruxolitinib in combination with ECP—in 18 patients with steroid refractory acute GVHD of lower GI-tract after ASCT. Endpoints of this study were complete remission of steroid refractory aGVHD, duration of response, incidence of severe infections, cytopenias and bleeding complications, as well as 2 year overall survival.

## Patients and methods

From June 2015 to February 2017, 18 patients (78% male, 22% female) with steroid refractory aGVHD of lower GI-tract who underwent an ASCT at the Department of Stem Cell Transplantation at the University Medical Center Hamburg-Eppendorf, Germany, due to different underlying diseases were treated with ruxolitinib and ECP (Table 1) and analyzed in this retrospective study. Median age was 58.5 years (*r*: 21–73), stem cell source was in all cases peripheral blood. Steroid refractoriness was defined as no improvement in 7 days or aggravation after 5 days of steroid treatment with 2 mg methylprednisolone/kg bodyweight.

**Table 1:** Patient and treatment characteristics.

Patient	Underlying disease	Sex	Age (years)	Donor source	Recipient CMV serostatus	Donor CMV serostatus	GVHD prophylaxis	Overall grade aGVHD at start Ruxolitinib/ECP	Additional organ involvement of aGVHD to lower GI-tract
1	MDS	Female	45	MUD	Negative	Negative	ATG CSA MMF	IV	None
2	PMF	Male	52	MRD	Positive	Negative	ATG CSA MMF	III	Skin and liver (grade I)
3	PMF	Male	71	MMUD 9/10	Positive	Positive	ATG CSA MMF	III	Skin and liver (grade II)
4	MM	Male	49	MRD	Negative	Positive	Post-Cy	IV	Liver (grade III)
5	PMF	Male	59	MUD	Positive	Positive	ATG CSA MMF	IV	Liver and upper GIT (grade I)
6	PMF	Female	67	MUD	Positive	Positive	ATG CSA MMF	IV	None
7	PMF	Male	67	MUD	Positive	Negative	Post-Cy	IV	Skin (grade I)
8	AML	Male	64	MUD	Negative	Negative	ATG CSA MMF	III	Liver (grade II)
9	ALL	Male	23	MUD	Positive	Positive	Post-Cy MMF Tacrolimus	IV	None
10	MDS	Male	55	MUD	Positive	Positive	ATG CSA MMF	III	None
11	MDS	Male	66	MMUD 9/10	Positive	Positive	ATG CSA MMF	III	None
12	ALL	Male	55	MMUD 9/10	Positive	Positive	Post-Cy MMF	III	Skin (grade I)
13	MDS	Male	72	MMUD 9/10	Positive	Negative	ATG CSA MMF	IV	None
14	ALL	Male	21	MMUD 9/10	Positive	Positive	Post-Cy CSA MMF	III	Skin (grade I)
15	AML	Female	58	MUD	Positive	Positive	ATG CSA MMF	IV	Upper GIT (grade I)
16	MDS	Male	30	MUD	Negative	Negative	ATG CSA MMF	IV	None
17	aCML	Female	63	MRD	Positive	Positive	ATG CSA MMF	III	Skin (grade III)
18	MDS	Male	73	MMUD 9/10	Positive	Positive	ATG CSA MMF	III	Skin and liver (grade II)

Grading sytem of aGVHD: according to Przepiorka et al. [14].

*CMV* cytomegalovirus, *MDS* myelodysplastic syndrome, *PMF* primary myelofibrosis, *MM* multiple myeloma, *AML* acute myeloid leukemia, *ALL* acute lymphoblastic leukemia, *aCML* atypical chronic myeloid leukemia, *MUD* matched unrelated donor, *MRD* matched related donor, *MMUD* mismatched unrelated donor, *ATG* rabbit antithymocyte globuline, *CSA* ciclosporin A, *MMF* mycophenalate mofetil, *Post-Cy* post-transplantationcyclophosphamide.

Some patients showed additional aGVHD of skin (*n* = 7), liver (*n* = 6), or upper GI-tract (*n* = 2) or a combination of them and all patients showed overall grade III (50%) or IV aGVHD (50%) at starting ruxolitinib or ECP therapy according to classification system of Przepiorka et al. [14].

The majority of patients (*n* = 15, 83%) received ruxolitinib before starting ECP with a median interval between starting ruxolitinib and initiation of ECP therapy of 19.7 days (*r*: 7–62). Median day of onset of any grade of any aGVHD after transplantation was day 30 (*r*: 11–198), median start of ruxolitinib or ECP was day 86.5 (*r*: 35–257), but prompt after fulfilling definition of steroid refractoriness. Dosage of methylprednisolone in all patients at initiation of ruxolitinib or ECP was 2 mg/kg bodyweight. Median duration of ruxolitinib therapy was 59.8 days (*r*: 14–192) with a median start dosage of 20 mg per day (2 × 10 mg; *r*: 10–20 mg). All patients started with two ECP treatments per week for two weeks with an individual reduction of treatment frequency, but in majority of patients, following ECP treatment was administered one time a week (*r*: 0.5–2.1 ECP treatments/week). Number of all ECP treatments differed from 2 to 71 treatments per patients (median: 20.5 treatments per patient), accordingly the duration of treatment differed from 0.4 to 23.4 months (median: 5.7 months). All ECP were performed with Therakos Cellex System.

All patients received an additional treatment with a calcineurin inhibitor to ruxolitinib/ECP therapy and/or methylprednisolone, 17 patients received additional mycophenolate mofetil. For analyzing main side effects of ruxolitinib and ECP therapy, cytomegalovirus (CMV) status of all patients as an indicator for infections and immune status of ten patients (lymphocyte count with CD4 + T lymphocyte and regulatory T cell count) were collected prior and after four weeks of combined treatment with ruxolitinib and ECP and 3–4 weeks after stopping ruxolitinib therapy. Regulatory T cell population was identified by sequential gating for SSC low and CD45 dim cells with CD3, CD4, and CD25 positivity. Fourteen patients (78%) were CMV-positive before ASCT and 12 patients (67%) received stem cells of CMV-positive donors. Three of these patients had high-risk constellation for suffering of CMV reactivation being CMV-positive before ASCT and receiving CMV-negative stem cells.

## Results

### Response to treatment and side effects

Overall response to the treatment with ruxolitinib, the combined immunosuppressive therapies and ECP in all patients were 56% (*n* = 10), including complete remission of 44% (*n* = 8) and partial remission of 11% (*n* = 2), while eight patients (44%) finally showed no response to the treatment. Response criteria were determined according to Przepiorka et al. [14].

The main reason for stopping either ruxolitinib or ECP therapy was cytopenia (*n* = 8, 44%), followed by receiving complete remission status (*n* = 6, 33%), severe infections or sepsis (*n* = 1, 6%), and in three patients (17%) no response to ruxolitinib and ECP therapy was the reason to stop the therapy (*n* = 2, 7%). In 33% of patients (*n* = 6), ruxolitinib and ECP therapy had to be discontinued for a few days (*r*: 1–10) due to different reasons (*n* = 2: infections, *n* = 2: vomiting and emesis, and *n* = 2 unknown reason), but all of the patients could restart with the therapy with same dosage as previously given. In the group of the eight patients who stopped ruxolitinib and ECP treatment due to cytopenia, three patients could recover platelet, hemoglobin, and leukocyte level within four weeks, two patients died in this cohort.

Analyzing all side effects during the therapy irrespective of grading, in 17 patients (94%) side effects associated with ruxolitinib and ECP could be observed, just in one patient no side effects have been documented (Table 2). As expected, the main side effect was cytopenia. Compared with the initial leukocyte, platelet and hemoglobin level, five patients (28%) developed worsening of anemia about one to two CTC grades (common toxicity criteria), seven patients (39%) worsening of the platelet count about one to three CTC grades, and in nine patients (50%) worsening of leukocyte count about one to three CTC grades was observed [15]. Granulocyte colony-stimulating factor was administered in eight of these patients. These data implicate the maximum of worsening cytopenia during ruxolitinib and ECP therapy.

**Table 2:** Response and side effects.

Patient	Response to treatment	Leukopenia	Anemia	Thrombopenia	Other side effects	CMV reactivation
1	CR	None	None	None	None	No
2	No response	None	CTC I	None	Fever CTC I	Yes
3	PR	CTC II	None	None	Fever CTC II	Yes
4	No response	None	None	None	None	No
5	PR	CTC I	CTC I	CTC I	Elevated CRP-level CTC II	Yes
6	CR	CTC II	None	None	None	Yes
7	No response	None	None	None	Elevated CRP-level CTC II	Yes
8	No response	CTC III	None	None	Fever CTC I elevated CRP-level CTC II	No
9	CR	CTC II	None	CTC III	Elevated CRP-level CTC II	Yes
10	No response	None	None	CTC II	Elevated CRP-level CTC I	Yes
11	CR	None	None	None	Fever CTC I elevated CRP-level CTC III	Yes
12	CR	CTC I	CTC I	None	None	Yes
13	No response	None	None	None	Fever CTC II elevated CRP-level CTC III sepsis	Yes
14	CR	CTC II	None	None	Fever CTC I	Yes
15	No response	CTC I	CTC I	None	Fever CTC II elevated CRP-level CTC I	No
16	No response	None	CTC II	CTC III	None	No
17	CR	CTC I	None	CTC II	None	No
18	CR	None	None	CTC I	Fever elevated CRP-level	Yes

Grading system of side effects: common terminology criteria for adverse events, version 5.0 [15].

Grading sytem of responding of aGVHD: according to Przepiorka et al. [14].

*CR* complete remission, *PR* partial remission, *CRP* C-reactive protein, *CTC* common toxicity criteria.

Increase of CRP level (C-reactive protein) over 50 mg/dl could be observed in nine patients (50%), three of them developed a sepsis. Fever was observed in eight patients (44%). CMV reactivation during ruxolitinib therapy as a parameter of infection occurred in 67% of cases (*n* = 12); all of the three patients mentioned in “Patients and methods” with high-risk constellation of host CMV positivity before ASCT, receiving CMV-negative donor stem cells were in this group of CMV reactivations after transplantation.

### Immune status and tapering of steroids

**Fig. 1:**
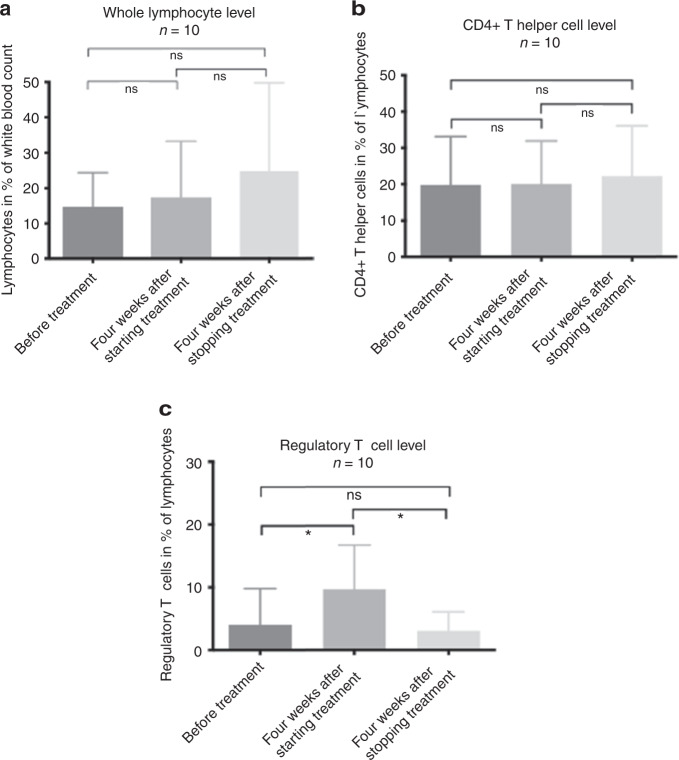
Comparison between pre-treatment, 4 weeks after treatment and 4 weeks after stopping treatment with Ruxolitinib and ECP. **a** Lymphocyte level in % of white blood count of ten patients before, during, and after treatment with ruxolitinib and ECP. No significant difference could be observed. **b** CD4+ T helper cell level in % of T cell count of ten patients before, during, and after treatment of ruxolitinib and ECP. No significant difference could be observed. **c** Regulatory T cell level in % of T cell count of ten patients before and after treatment of ruxolitinib and ECP. Significant difference could be observed between time point before starting therapy and during therapy and between stopping therapy and during therapy (*p* = 0.02, respectively).

**Fig. 2:**
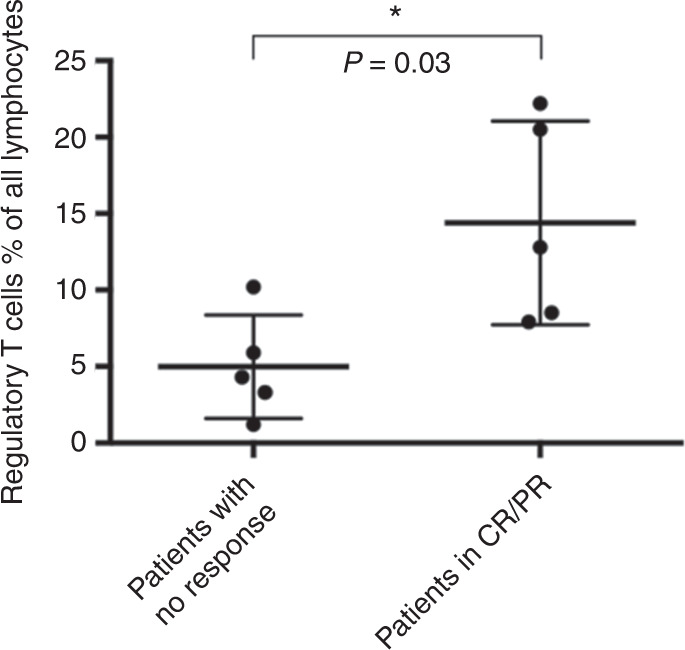
Comparison of regulatory T cell level between patients with no response or CR/PR four weeks after starting treatment.

Comparing immune reconstitution—including whole lymphocyte count, CD4+ T helper cell and regulatory T cell count—of the ten patients from whom samples were collected before starting combined ruxolitinib and ECP therapy, four weeks after starting treatment and 3–4 weeks after stopping the therapy, we could not observe significant changes neither in whole lymphocyte count nor in count of CD4+ T helper cells comparing time point before, during and after stopping the therapy. Interestingly, regulatory T cells significantly increased during combined ruxolitinib/ECP treatment compared with regulatory T cell count before treatment (*p* = 0.02) and after stopping treatment, regulatory T cell count decreased again (*p* = 0.02; Fig. 1). In addition, we could observe high regulatory T cell levels especially in those patients, who reached CR or PR under the treatment of ruxolitinib and ECP (*p* = 0.03; Fig. 2).

Another important aspect during therapy with ruxolitinib and ECP is tapering of steroids in the steroid refractory setting. In our cohort to avoid harmful effects of steroid treatment, tapering of steroids could be performed rapidly with a maximum reduction time of 7 days for reducing to half of the dosage (median: 2 days). Reduction to a quarter of initial dosage of methylprednisolone was reached in a median time of 6 days (*r*: 1–12 days). Complete stopping of steroids could be reached in a median time of 27 days after starting ruxolitinib and ECP therapy (*r*: 12–63, Fig. 3).

**Fig. 3:**
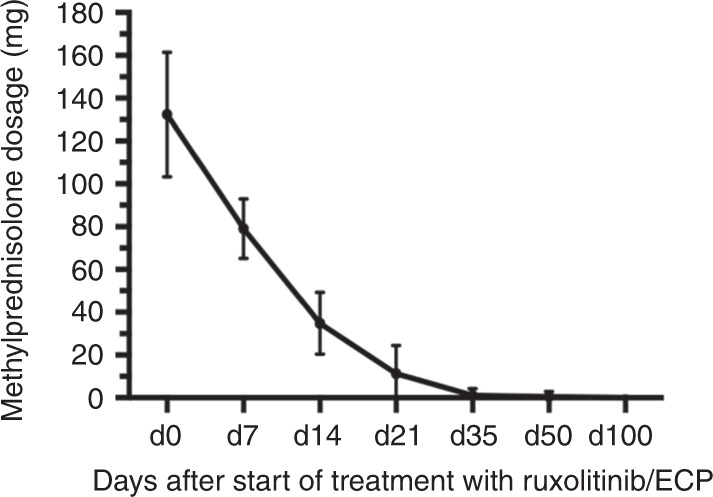
Reduction of methylprednisolon in mg after start of treatment with ruxolitinib/ECP. Reduction of steroids under the treatment with ruxolitinib/ECP.

### Overall survival and long-term follow-up

**Fig. 4:**
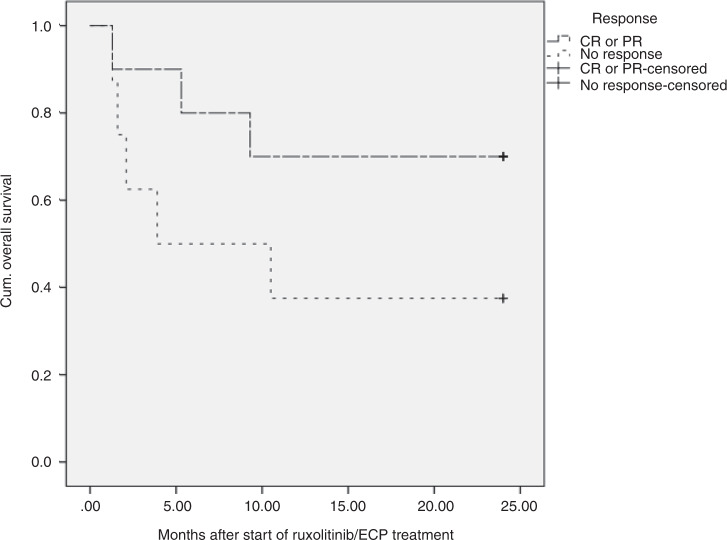
Kaplan–Meier survival curve for 2 year estimated overall survival after start of ruxolitinib and ECP therapy. 38% of patients who did not respond to the treatment survived after 2 years (median survival time: 11.4 months). In the cohort of patients who reached CR/PR under the treatment, 70% survived after 2 years (median survival time: 18.4).

Two year estimated overall survival of all patients after starting with ruxolitinib and ECP was 56% (*n* = 10) with a median estimated overall survival of 15.3 months. Comparing patients who did not respond to the therapy to those who reached CR or PR, a lower overall survival of 38% in non-responders (median overall survival: 11.4 months) could be observed than in the patients with CR/PR with an estimated overall survival of 70% (median overall survival: 18.4 months, *p* = 0.1; Fig. 4). Eight patients died during the therapy, three because of relapse of underlying disease, one patient due to severe therapy refractory aGVHD of lower GI-tract, and four due to infection complications in aGVHD refractory setting.

After a median follow up of 886.9 (*r*: 736–1021) days of the ten surviving patients after 2 years after starting ruxolitinib and ECP therapy, five patients had continuous CR of aGVHD, four had continuous PR, and just one patient had a relapse of aGVHD. Overall, cGVHD developed in 67% (*n* = 12). Two patients developed severe cGVHD, four patients moderate, and the majority of six patients suffered from mild cGVHD according to NIH classification [16]. Just two patients developed quiescent cGVHD, the majority of ten patients had progressive cGVHD during/after treatment of ruxolitinib and ECP. Two patients with cGVHD and the patient with relapse of aGVHD restarted with ruxolitinib and ECP treatment due to severe fourth grade cGVHD, all of them had a partial remission.

## Discussion

In this single-center study, only patients with severe lower GI steroid refractory aGVHD—defined by no improvement in 7 days or aggravation after 5 days of steroid treatment with 2 mg methylprednisolone/kg bodyweight—were included. All patients were treated in a combined approach using ruxolitinib and ECP in order to increase the rate of responders and to show an increase of regulatory T cells. Treatment of severe steroid refractory aGVHD is still challenging in clinical practice. Finding a balance between therapy and side effects in this special cohort of patients is the main difficulty. On the one hand, therapeutic strategies with high efficacy are necessary to reduce symptoms and repress this complication after ASCT. On the other hand, it is important to keep the immunosuppressive effect as low as possible in order to reduce severe infections caused by an affected host immune system and to maintain the graft-versus-leukemia effect. Ruxolitinib and ECP are well known to show efficacy in prevention and treatment of acute and cGVHD in preclinical and clinical settings [6, 9–11, 13]. Nevertheless, for steroid refractory patients with severe aGVHD the prognosis remains poor and recommendations for second- and third-line therapeutic approaches are controversial.

Our cohort represents severely affected patients with steroid refractory acute GVHD overall grades III or IV. Combining ruxolitinib and ECP, we could show a high rate of complete remission of 44%, a partial remission rate of 11%, and an encouraging 2 year overall survival of 70% in those patients who could reach CR or PR. In previous study, ECP alone could show high rates of partial remission, but very low rates of complete remission in steroid refractory aGVHD of 4.8% [13]. However, treatment with ruxolitinib without ECP in steroid refractory aGVHD overall grade III or IV could show comparable results with our cohort with a complete response rate of 46.3% in previous study [11]. Side effects were comparable with those of previous published data [11, 13] with remarkable no grade IV cytopenias in our cohort despite combining these two therapies. According to this, especially the rate of patients suffering from severe infections was low with four patients who died due to infection-associated complications all in aGVHD refractory setting. This could be explained by early tapering of steroids after starting the combination therapy of ruxolitinib and ECP, rapidly reducing and avoiding the additional side effects of steroid therapy. However, CMV reactivation was represented with a high rate of 67%, but ruxolitinib and ECP did not affect the efficacy of following anti-viral treatment.

Analyzing immune reconstitution in selected patients we could show that regulatory T cells increased during the treatment of ruxolitinib and ECP, an effect which strengthen the assumption of potential efficacy of these two approaches for treating severe steroid refractory aGVHD, because it is well described that regulatory T cells can prevent graft-versus-host disease without negatively affecting graft-versus-leukemia effect [6, 15, 17]. In aGVHD mouse model, the same effect could be shown: mice treated with ruxolitinib showed an increased level of regulatory T cells in blood, spleen, and aGVHD-affected ileum and colon [6]. Functioning as a suppressor of autoreactive lymphocytes which sustain acute and cGVHD, regulatory T cells can facilitate long-lasting immune tolerance [6, 15, 17, 18]. Here, we could show for the first time that higher levels of regulatory T cells can be measured during combined treatment of ruxolitinib and ECP in clinical setting especially in those patients who received CR or PR during the treatment of ruxolitinib and ECP, which elucidates the effect of regulatory T cells in the pathogenesis of aGVHD and underlines the efficacy of this combined treatment approach. To figure out which therapeutic approach affects regulatory T cell level most—ECP or ruxolitinib alone—further prospective studies are necessary, comparing both therapeutic strategies in a matched patient group.

It is, in addition, remarkable that just one patient developed a relapse of aGVHD after stopping the combined therapy, perhaps underlying long-lasting effects of regulatory T cells induced by ruxolitinib therapy. However, developing cGVHD still remains a problem: high percentage of 70% developed cGVHD later on.

In conclusion, we could show that combining ruxolitinib and ECP in severe steroid refractory aGVHD after ASCT shows comparatively high efficacy and acceptable tolerability, allowing rapid tapering of steroids with a low rate of severe infections.

## References

[1] Jaglowski SM, Devine SM (2014). Graft-versus-host disease: why have we not made more progress?. Curr Opin Hematol.

[2] Zhang L, Yu J, Wei W (2018). Advance in targeted immunotherapy for graft-versus-host disease. Front Immunol.

[3] Cesen Mazic M, Girandon L, Knezevic M, Avcin SL, Jazbec J (2018). Treatment of severe steroid-refractory acute-graft-vs.-host disease with mesenchymal stem cells-single center experience. Front Bioeng Biotechnol.

[4] Deeg HJ (2007). How I treat refractory acute GVHD. Blood.

[5] Bousoik E, Montazeri Aliabadi H (2018). “Do We Know Jack” About JAK? A closer look at JAK/STAT signaling pathway. Front Oncol.

[6] Spoerl S, Mathew NR, Bscheider M, Schmitt-Graeff A, Chen S, Mueller T (2014). Activity of therapeutic JAK 1/2 blockade in graft-versus-host disease. Blood.

[7] Verstovsek S, Mesa RA, Gotlib J, Levy RS, Gupta V, DiPersio JF (2012). A double-blind, placebo-controlled trial of ruxolitinib for myelofibrosis. N Engl J Med.

[8] Zeiser R, Blazar BR (2017). Acute graft-versus-host disease—biologic process, prevention, and therapy. N Engl J Med.

[9] Kroger N, Shahnaz Syed Abd Kadir S, Zabelina T, Badbaran A, Christopeit M, Ayuk F (2018). Peritransplantation ruxolitinib prevents acute graft-versus-host disease in patients with myelofibrosis undergoing allogenic stem cell transplantation. Biol Blood Marrow Transpl.

[10] Schroeder MA, Choi J, Staser K, DiPersio JF (2018). The role of janus kinase signaling in graft-versus-host disease and graft versus leukemia. Biol Blood Marrow Transpl.

[11] Zeiser R, Burchert A, Lengerke C, Verbeek M, Maas-Bauer K, Metzelder SK (2015). Ruxolitinib in corticosteroid-refractory graft-versus-host disease after allogeneic stem cell transplantation: a multicenter survey. Leukemia.

[12] Saeed I, McLornan D, Harrison CN (2017). Managing side effects of JAK inhibitors for myelofibrosis in clinical practice. Expert Rev Hematol.

[13] Sakellari I, Gavriilaki E, Batsis I, Mallouri D, Panteliadou AK, Lazaridou A (2018). Favorable impact of extracorporeal photopheresis in acute and chronic graft versus host disease: prospective single-center study. J Clin Apher.

[14] Przepiorka D, Weisdorf D, Martin P, Klingemann HG, Beatty P, Hows J (1995). 1994 consensus conference on acute GVHD grading. Bone Marrow Transpl.

[15] Cao J, Chen C, Zeng L, Li L, Li Z, Xu K (2010). Engineered regulatory T cells prevent graft-versus-host disease while sparing the graft-versus-leukemia effect after bone marrow transplantation. Leukemia Res.

[16] Lee S (2017). Classification systems for chronic graft-versus-host disease. Blood.

[17] Carniti C, Gimondi S, Vendramin A, Recordati C, Confalonieri D, Bermema A (2015). Pharmacologic Inhibition of JAK1/JAK2 signaling reduces experimental murine acute GVHD while preserving GVT effects. Clin Cancer Res.

[18] Koreth J, Matsuoka K, Kim HT, McDonough SM, Bindra B, Alyea EP (2011). Interleukin-2 and regulatory T cells in graft-versus-host disease. N Engl J Med.

