# Correction: Ruxolitinib plus extracorporeal photopheresis (ECP) for steroid refractory acute graft-versus-host disease of lower GI-tract after allogeneic stem cell transplantation leads to increased regulatory T cell level

**DOI:** 10.1038/s41409-021-01425-4

**Published:** 2021-08-19

**Authors:** Franziska Modemann, Francis Ayuk, Christine Wolschke, Maximilian Christopeit, Dietlinde Janson, Ute-Marie von Pein, Nicolaus Kröger

**Affiliations:** 1 Department of Stem Cell Transplantation, University Medical Center Hamburg-Eppendorf, Hamburg, Germany; 2 Department of Oncology and Hematology, University Medical Center Hamburg-Eppendorf, Hamburg, Germany

**Keywords:** Stem-cell research, Disease-free survival

Correction to: *Bone Marrow Transplantation* 10.1038/s41409-020-0952-z, published online 23 May 2020

The article “Ruxolitinib plus extracorporeal photopheresis (ECP) for steroid refractory acute graft-versus-host disease of lower GI-tract after allogeneic stem cell transplantation leads to increased regulatory T cell level”, written by Franziska Modemann, Francis Ayuk, Christine Wolschke, Maximilian Christopeit, Dietlinde Janson, Ute-Marie von Pein, Nicolaus Kröger, was originally published Online First without Open Access. After publication in volume 55, issue 12, page 2286–2293, the author decided to opt for Open Choice and to make the article an Open Access publication. Therefore, the copyright of the article has been changed to © The Author(s) 2020 and the article is forthwith distributed under the terms of the Creative Commons Attribution 4.0 International License, which permits use, sharing, adaptation, distribution and reproduction in any medium or format, as long as you give appropriate credit to the original author(s) and the source, provide a link to the Creative Commons license, and indicate if changes were made. The images or other third party material in this article are included in the article’s Creative Commons license, unless indicated otherwise in a credit line to the material. If material is not included in the article’s Creative Commons license and your intended use is not permitted by statutory regulation or exceeds the permitted use, you will need to obtain permission directly from the copyright holder. To view a copy of this license, visit http://creativecommons.org/licenses/by/4.0. Open access funding is enabled and organized by Projekt DEAL.

